# The miR-486-5p plays a causative role in prostate cancer through negative regulation of multiple tumor suppressor pathways

**DOI:** 10.18632/oncotarget.20427

**Published:** 2017-08-24

**Authors:** Yang Yang, Changwei Ji, Suhan Guo, Xin Su, Xiaozhi Zhao, Shiwei Zhang, Guangxiang Liu, Xuefeng Qiu, Qing Zhang, Hongqian Guo, Huimei Chen

**Affiliations:** ^1^ Department of Urology, Drum Tower Hospital Affiliated with Nanjing University School of Medicine, Institute of Urology, Nanjing University, Nanjing 210008, China; ^2^ School of Medicine, Nanjing University, Nanjing 210093, China; ^3^ Jiangsu Key Laboratory of Molecular Medicine, Nanjing 210002, China; ^4^ School of Public Health, Nanjing Medical University, Nanjing 211166, China; ^5^ Center of Drug Discovery, State Key Laboratory of Natural Medicines, China Pharmaceutical University, Nanjing 211198, China

**Keywords:** prostate cancer, miR-486-5p, cancer driver, oncogenic pathway, transcription factor

## Abstract

MicroRNAs have been broadly implicated in cancer, but their exact function and mechanism in carcinogenesis remain poorly understood. Aberrant miR-486-5p expression is frequently found in human cancers. Here we showed a significant overexpression of miR-486-5p in prostate cancer compared with that in normal tissue and cells, and we proposed that altered expression of miR-486-5p in the prostate contributed to prostate cancer. Firstly, miR-486-5p inhibition expression reduced prostate cancercell proliferation, migration, and colonization *in vitro* and prostate tumor development *in vivo*. Moreover, we integrated RNA sequencing and target genes prediction, and systemically identified miR-486-5p candidate target genes. We conducted an experiment verifying that miR-486-5p drives tumorigenesis by directly targeting multiple negative regulators, which were involved in PTEN/PI3K/Akt, FOXO, and TGF-b/Smad2 signaling. Finally, we demonstrated that hypoxia-inducible factor-1a and TCF-12 are located at the miR-486-5p promoter, which stimulates the transcription of miR-486-5p itself. Collectively, our findings unveil miR-486-5p as a powerful prostate cancer driver that coordinates the activation of multiple oncogenic pathways and demonstrates some stimulators, which mediate the miR-486-5p signaling pathway and may be targeted for therapy.

## INTRODUCTION

Prostate cancer (Pca) is the most common malignancy in men and one of the leading causes of cancer death in United State [1]. By the time a prostate cancer is diagnosed, it often comprises millions to billions of cells carrying genetic and expression alterations that initiated the malignant transformation. Some alterations are strong and causal ‘drivers’ that confer selective growth advantages to cancer cells, but most are incidental ‘passengers’ that have been added during the cancer's life history [2]. The key challenge in cancer research is to distinguish the drivers and contributors from the passengers. Assessing the contribution of individual alterations to cancer and establishing their causality in cancer are essential to the development of cancer therapeutics. Therapies targeted toward BRCA2 have proven to be of clinical benefit in selected subgroups of patients with prostate cancers [3]. More drivers could eventually be leveraged for therapeutic purposes.

MicroRNAs (miRNAs) are small, noncoding, single-stranded RNAs of ∼22 nucleotides that negatively regulate gene expression at the posttranscriptional level, primarily through base pairing to the 3′ untranslated region (UTR) of target mRNAs [4, 5]. Growing evidence indicates that miRNAs control basic cell functions, ranging from proliferation to apoptosis. Recently, an altered expression of miRNAs has been observed in most cancers [6]. Some of them have been demonstrated as drivers for certain cancers, which provide evidence of the development of miRNA-based therapeutics [7]. Gene expression profiling of human prostate cancers has revealed various miRNA expression signatures [8, 9].

Primarily, we observed an up-regulated miR-486-5p expression in the cases of prostate cancers. Due to a lack of a detailed investigation, an altered expression of miR-486-5p has also been described in published array data. Two recent investigations identified miR-486 as a causal driver for lung cancer [10, 11]. Investigators have identified a few potential targets for miR-486-5p, including OLFM4 [12], SIRT1 [13], and PIM-1 [14] as well as the tumor suppressors, PTEN [15] and FOXO1 [15, 16]. The investigators also found components of the insulin growth factor (IGF) signaling, including the insulin-like growth factor 1 (IGF1), the IGF1 receptor (IGF1R), and phosphoinositide-3-kinase, regulatory subunit 1 (alpha) (PIK3R1 or p85a) [16].

It is important to understand how critical miR-486-5p is in the development of prostate cancer. The mechanistic role for miR-486-5p as either an oncogene or tumor suppressor, particularly in prostate cancer, remains largely unknown. In-depth understanding of these questions will determine whether targeting miR-486-5p, its mediators, or the downstream pathways has any therapeutic value for prostate cancer. In the present study, we have identified miR-486-5p as a key miRNA that drives prostate cancers, through negatively regulated multiple tumor suppressor pathways. This paper also demonstrates the regulator factors involved in miR-486-5p transcription.

## RESULTS

### MiR-486-5p Is up-regulated in PCa

**Figure 1:**
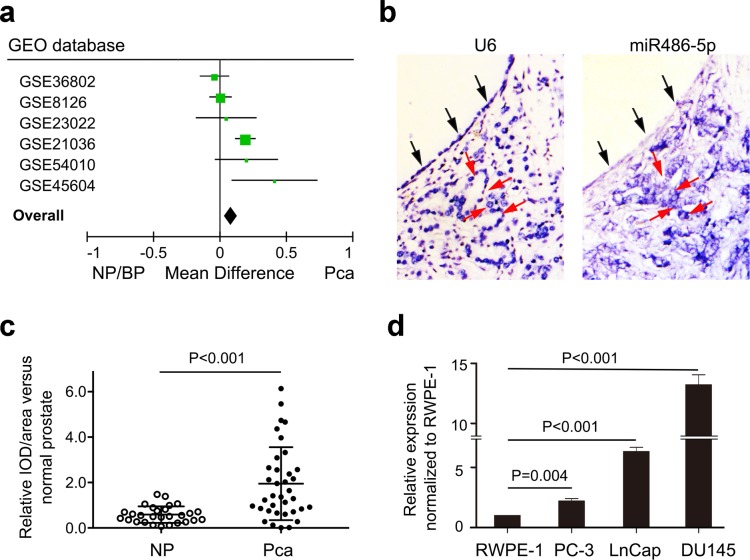
Up-regulation of miR-486-5p expression in prostate cancer patients and cell lines **(a)** Meta-analysis of the six groups profiles of microRNA in prostate cancer from GEO datasets. **(b)** *In situ* hybridization (ISH) showed overexpression of miR-486-5p in cancer tissues (red arrow) compared to benign tissue (black arrow). **(c)** The statistic analysis of miR-486-5p relative expression in ISH. The relative value was calculated by the Image-Pro Plus software. NP, normal prostate tissue; Pca, prostate cancer tissue. **(d)** Up-regulation of miR-486-5p expression was validated in three prostate cancer cell lines by real-time PCR using U6 as control.

We analyzed miR-486-5p expression in 250 PCa tissues and 97 control tissues using mircroRNA expression profiles from a GEO database (Supplementary Table 4). Meta-analysis showed that miR-486-5p was up-regulated in prostate tumors when compared with controls (P = 0.0005; Figure 1a). *In situ* hybridization of 23 PCa cases were further used and confirmed as an overt increase in the levels of miR-486-5p as compared to the adjacent normal prostate tissues (NP) (P < 0.001; Figure 1b & 1c). But, no significant difference was observed between different Gleason scores (P>0.05; Supplementary Table 1).

**Figure 2:**
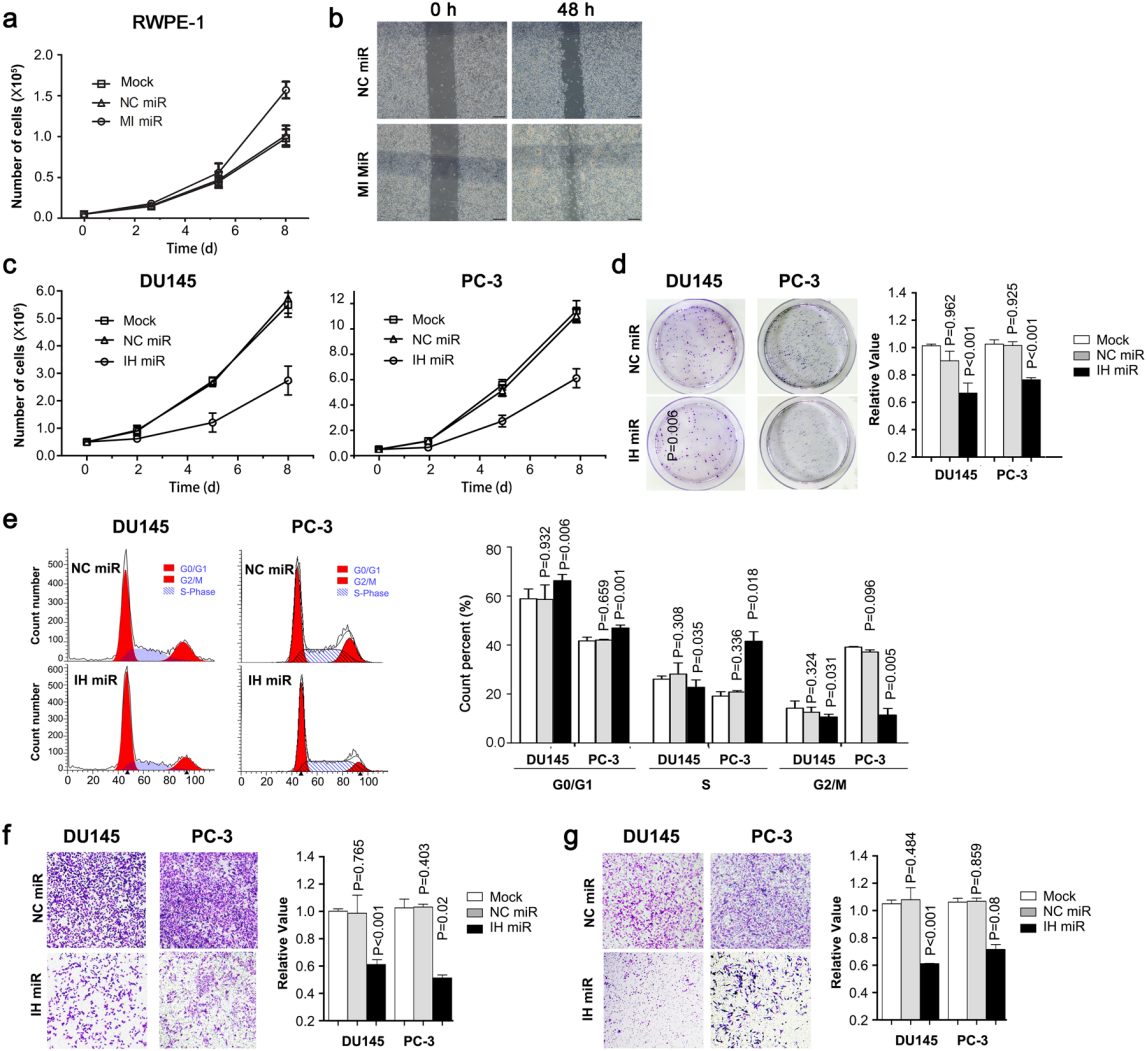
Ectopic expression of miR-486-5p affects cell growth, colony formation, cell cycle, cell migration and invasiveness **(a)** RWPE-1 cells were transfected with miR-486-5p mimics (MI-miR), negative control (NC-miR) or nothing (mock) and viable cells were counted with trypan blue to measure cell growth. **(b)** RWPE-1 cell migration ability was measured by a wound-healing assay. The relative percent of wound closure was calculated at 48 h before the complete would closure. **(c)** Du145 or PC-3 cells were transfected with miR-486-5p inhibitor (IH miR), inhibitor negative control (NC miR) or nothing (mock) and viable cells were counted with trypan blue to measure cell growth. **(d)** Cell colonies were stained with crystal violet solution, dissolved in 33% ethanoic acid buffer and then measure OD590nm for quantification. **(e)** Flow cytometric distribution of Du145 and PC-3 cells transfected with IH miR or NC miR. **(f** and **g)** Du145 and PC-3 cells transfected with IH miR or NC miR were seeded in commerical transwell plate and incubated at 37°C for 48 h. The migrated cells were stained with crystal violet solution, dissolved in 33% ethanoic acid buffer, and then measured at OD590nm for quantification.

Real-time PCR showed a consistent upregulation of miR-486-5p in each of three prostate cancer cell lines, DU145 (p < 0.001), PC-3 (p = 0.004), and LnCap (p < 0.001), with respect to RWPE-1 cell line (Figure 1d). RWPE-1cell line is the widely used normal prostate epithelial cells. The overexpression of miR-486-5p in RWPE-1cell line promoted the cell proliferation, with assessed by cell count in MI-miR group than that in NC-miR and mock groups (Figure 2a). The MI-miR group also showed an enhanced migration capacity compared with the NC-miR group (Figure 2b).

### Inhibition of miR-486-5p protects PCa *in vitro*

Given the high expression in PCa, we hypothesized that miR-486-5p may function as a carcinogen in the prostate. We transiently transfected miR-486-5p inhibitor into two independent PCa cell lines, DU145 and PC-3 and the levels of miR-486-5p were restored to a low level in both cells and maintained for at least six days (Supplementary Figure 1).

The inhibition of miR-486-5p in both Du145 and PC-3 cell lines led to a considerable reduction in cellular proliferation, as assessed by the cell count as shown in Figure 2c. A lower colony formation was also observed in both cell lines with the decreased levels of miR-486-5p, when compared with respective controls (Figure 2d). The decreased miR-486-5p significantly increased the percentage of PCa cells in the phase of sub-G0 and G1, suggesting a restraining role on cell-cycle progression (Figure 2e). Moreover, Du145 and PC-3 cells infected with the miR-486-5p inhibitor revealed a depressed migration capacity in standard medium, as well a decreased invasion and motility in a cancer fibroblast-conditioned medium (Figure 2f & 2g). These data established a direct correlation between miR-486-5p booming and increased proliferation and migration of Pca.

### Loss of miR-486-5p suppresses tumorigenicity *in vivo*

**Figure 3:**
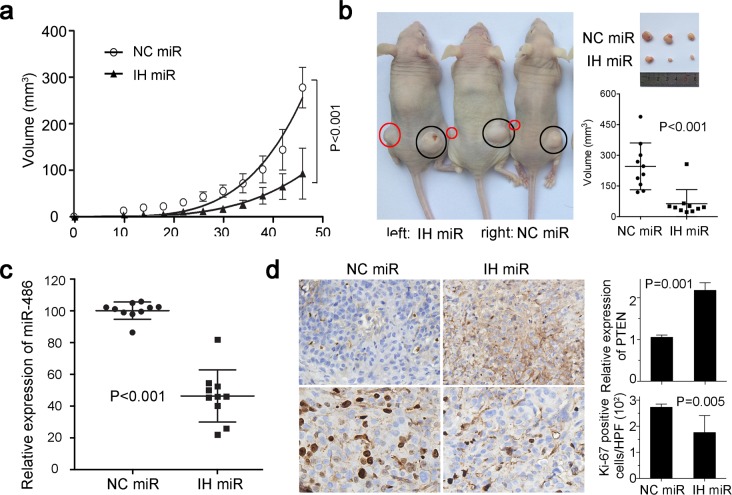
MiR-486-5p affects tumor growth in xenograft model **(a)** Tumor growth in nude mice subcutaneous injected into the two flanks with Du145 cells transfected with IH miR or NC miR. Datas are represented as mean ± SD (n=10). The curves represented the trend of the tumor size increase. **(b)** Comparison of tumor engraftment sizes in nude mice injected with Du145 cells transfected with IH miR or NC miR. The tumor were marked by the red and black circles on the nud mice. **(c)** MiR-486-5p expression in xenografts with IH miR or NC miR was measured by qPCR. **(d)** Immunohistochemistry showed PTEN and Ki67 expressions in xenografts with IH miR or NC miR.

We further investigated whether the function loss of miR-486-5p would suppress the tumorigenic ability of the Du145 cell line in nude-mice. The cells infected with inhibitor negative control produced fast-growing tumors in 4-week-old male nude-mice (Figure 3a). However, the inhibitor significantly induced a lower level of miR-486-5p cells *in situ* tumor, and caused a substantial reduction in tumor volume nearly 50 days after injection (Figure 3b & 3c). PTEN is usually mutated, downregulated or dysfunctional in Pca [17], but a higher level of PTEN was detected in the tumor transfected with miR-486-5p inhibitor *in vivo*. At the same time, a low level of Ki67 was maintained *in vivo* after being transplanted in mice for about 50 days (Figure 3d). These *in vivo* data confirmed that miR-486-5p functions as a carcinogen for PCa.

### Screening targets of miR-486-5p in PCa

**Figure 4:**
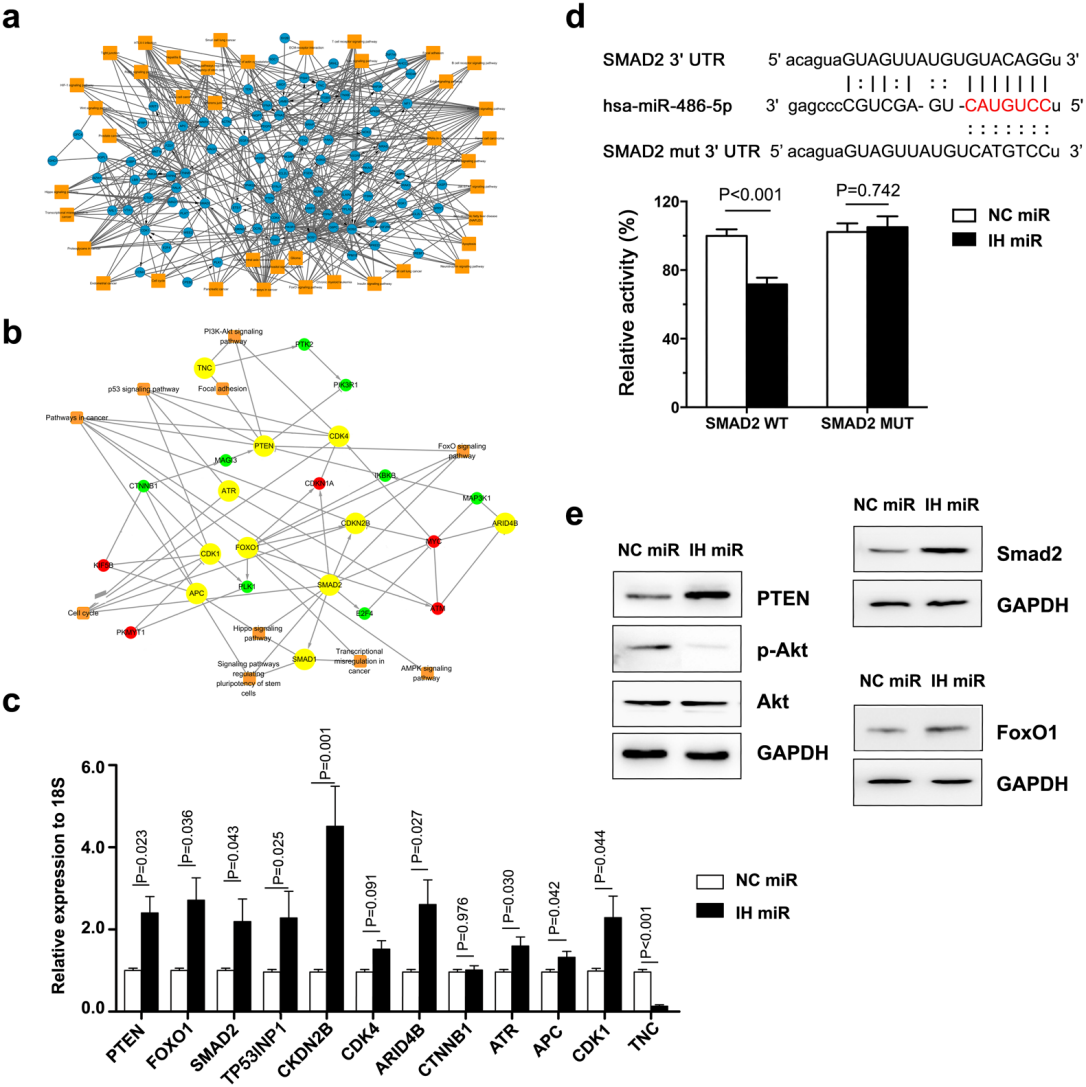
Target genes of miR-486-5p were screened and analysed **(a)** RNA sequencing showed that regulated genes were involed in numberous pathways, including PTEN/PI3K/Akt, FOXO1 and TGFβ/SMAD2 pathways. **(b)** After analyseing the results of RNA sequencing and target bioinformatic prediction, we focused on 11 genes which were considered as key targets and also involved in the preferential pathways. **(c)** The regulation of the 11 possible “driver” genes was validated in DU145 cell lines by real-time PCR. **(d)** SMAD2 3′UTR contains predicted miR-486-5p binding sites. The figure show alignment of miR-486-5p with SMAD2 3′UTR and the dotted line indicate the mutagenesis nucleotides. Luciferase reporter constructs containing wild-type or mutated SMAD2 3′UTR was cotransfected with IH miR and NC miR into Du145 cells. Relative firefly luciferase expression was normalized to Renilla luciferase. **(e)** Effect of miR-486-5p on PTEN/PI3K/Akt, FOXO1 and TGFβ/SMAD2 pathways. Du145 cells were transfected with IH miR and NC miR for 72 h, total proteins were prepared for Western blot.

We integrated mRNA expression profiling with bioinformatic predictions to explore the molecular pathways underlying prostate carcinogenesis derived by miR-486-5p. Then the mRNA microarray showed a list of 895 markedly up-regulated genes (fold change > 1.5, FDR< 0.05) in IH-miR-486-5p treated DU145 cells, compared to NC-miR treated cells. KEGG analysis indicated that the regulated genes were significantly involved in several preferential pathways, including PTEN/PI3K, FOXO1 and TGFβ/SMAD2 (all p < 0.05, Figure 4a). We further screened a candidate list for miR-486-5p targets, which were produced by all three prediction algorithms, PicTar, TargetScan and miRanda (microRNA.org). Since these candidates interact with each other, we focused on the key targets, which related to more than five other predicted genes under KEGG analysis (Figure 4b).

Finally, we identified 12 genes as the possible “driver” targets of miR-486-5p, which were considered as key targets and involved in the preferential pathways. These genes mainly contributed to the cell cycle, forkhead box O (FOXO) pathway, phosphatidylinositol-3 kinase (PI3K) pathway and transforming growth factor beta (TGFβ pathway).

### MiR-486-5p targets multiple pathways for PCa

To verify the targets identified, we evaluated the mRNA expression level of the 12 candidate genes (Figure 4c). PTEN and FOXO1 were previously reported to be the target of miR-486-5p in neonatal rat cardiomyocytes [15], chronic antibody-mediated rejection in kidney transplantation [18] and chronic myeloid leukemia [19], together or singlarly. MiR-486-5p inhibition resulted in increased PTEN, FOXO1. In addition, the function loss of miR-486-5p also increased the expression of PTEN of the PCa cell lines Du145 in nude-mice (Figure 3d).

Few test results showed that miR-486-5p directly targeted Smad2, and the 3′ untranslated regions (UTR) of Smad2 mRNA harbored sequences complementary to the miR-486 seed sequence. To confirm such a direct effect, both the wild-type (WT) and mutant (MUT) 3′ UTR sequences of Smad2 were synthesized and cloned. A consistent reduction in luciferase activity occurred for the 3′ UTR of Smad2 by miR-486-5p (Figure 4d). But, co-transfection of miR-486-5p with the MUT forms of the 3′ UTRs resulted in no significant change in luciferase activity. These data support the notion that Smad2 serves as a novel direct target of miR-486-5p in prostate cells, as well the miRNA/ target 3′ UTR specificity.

### HIF-1a and TCF12 stimulate miR-486-5p expression

**Figure 5:**
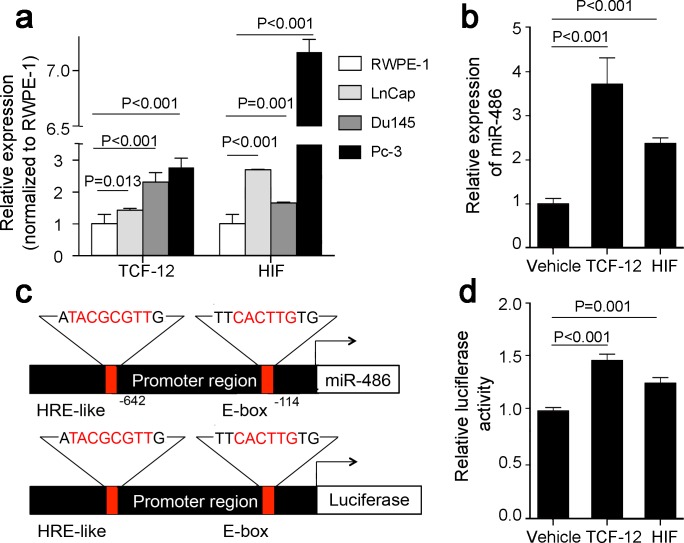
HIF1a and TCF12 mediate miR-486-5p induction in prostate cancer **(a)** Up-regulation of HIF1a and TCF12 was validated in three prostate cancer cell lines by real-time PCR. **(b)** Overexpressed HIF1a and TCF12 inducted overexpression of miR-486-5p in Du145 cells using vehicle plasmid as control. **(c)** Upper panel: miR-6486-5p promoter region harboring HRE binding site and E-box. Lower panels: Promoter reporter vectors containing the miR-486-5p promoter with HRE binding site and E-box upstream of luciferase gene. The core DNA binding sequence of HRE binding site and E-box is highlighted in red. **(d)** The luciferase promoter reporter vector containing the promoter region sequence was transfected into HeLa cells. Overexpressing the TCF12 and HIF-1a caused a significant induction of luciferase activity.

MiR-486-5p was controlled by an alternative promoter within intron 40 of the Ankyrin 1 gene, which also regulated the small ANK1 (sANK1) transcript [15]. MiR-486-5p and sANK1 could be co-regulated though the same promotor. Sequence analysis showed that the upstream of the promotor contains an HRE-like site and an E-box site (Figure 5c), which were hypoxia-inducible factor-1a (HIF-1a) and transcription factor 12 (TCF12) binding sites, respectively. This indicates HIF-1a and TCF12 may stimulate the promotor of sANK1 and miR-486-5p.

QRT-PCR showed a trend toward prostate cancer cell lines having higher HIF-1a and TCF12 mRNA expression than the normal prostate cell line (p < 0.05, Figure 5a). Overexpressed HIF-1a and TCF12, according the expression of miR-486-5p, was upregulated in the Du145 cell (Figure 5b). Furthermore, we cloned the promoter region sequence containing these two sites into a luciferase promoter reporter vector (Figure 5c), which were then transfected into HeLa cells to determine its promoter activity. It was determined that overexpression of TCF12 and HIF-1a caused a significant induction of luciferase activity (Figure 5d). These findings suggested that TCF12 and HIF-1a indeed bind to the upstream of promoter and positively activated miR-486-5p expression.

## DISCUSSION

Prostate cancer remains the most frequently diagnosed cancer among men in North America, and researching the pathogenesis of prostate cancer has become a top priority in the cancer-research field. This study shows that elevated miR-486-5p expression is sufficient to drive prostate cancer. It revealed that miR-486-5p drives prostate cancer by suppressing the expression of multiple inhibitors of the cancer pathways reducing both proliferative and migratory capacity, as well as inducing apoptosis in prostate cancer cell lines. Specifically, the activation of TCF12 and HIF-1a under these conditions leads to the transcriptional activation of miR-486-5p. To the best of our knowledge, the findings presented herein are the first to establish miR-486-5p as a powerful prostate cancer driver, and therefore, a valid target for cancer therapeutics. The research presented herein places miR-486-5p in the pantheon of prominent cancer genes that includes PTEN and FOXO1.

The ubiquitous nature of miR-486-5p gene amplification and overexpression in human cancers indicate a possible connection to other cancer types as well. In lung cancer, miR-486-5p has emerged as a noninvasive biomarker after being detected in both the plasma and sputum [20, 21]. However, miR-486-5p functions as a potent tumor suppressor in lung cancer [10]. This phenomenon could also be observed in other microRNAs. MiR-214 induces cell survival and cisplatin resistance by targeting PTEN in ovarian cancer [22] while MiR-214 targets the β-catenin pathway to suppress invasion, stem-like traits, and the recurrence of human hepatocellular carcinoma [23]. This warrants a comprehensive investigation of miR-486-5p in additional cancer models in the future. The expression of miR-486-5p was up-regulated in prostate cancer but differed from different cell lines so as to the different patients. There was no significant difference among different risk groups. Whether the mode of action had specificity in different subtype cancer cell line was not very clear.

This study fur ther placed miR-486-5p in the pantheon of prominent cancer genes. While bioinformatic algorithms have predicted thousands of potential target genes for miR-486-5p, only a few of them (*i.e.,* PTEN, FOXO1) have been experimentally validated in various cellular contexts. It was unclear which ones of the predicted targets are direct targets and whether miR-486-5p regulates various sets of target genes in different cellular contexts. Our mRNA array under different miR-486-5p expression analysis identified conserved target genes. Since these candidates interact with other genes, this study identified the “driver” genes that lead to the elucidation of molecular mechanisms underlying the role of miR-486-5p in prostate cancer. We only focused on the preferentially regulated pathways under the function loss of miR-486-5p in prostate cells and node targets under computational prediction. That process seemed an efficient protocol, with which we identified five mostly possible target pathways of miR-486-5p in PCa. We further experimentally validated that miR-486-5p directly suppresses the expression of PTEN, FOXO1 and SMAD2, and then contributes to prostate cancers, which makes it subject to complex regulation by miRNAs.

PTEN and FOXO1, which serve as negative components of phosphoinositide-3-kinase (PI3K)/Akt signaling have a direct function in proliferation suppression. This is well exemplified by direct regulation of PTEN and FOXO1 by miR-486-5p in polycystic ovary syndrome, sebaceous carcinoma and chronic kidney disease [16, 24, 25]. Moreover, the most identified miR-486-5p targets in prostate cells are broadly expressed genes, implying that miR-486-5p regulates a common set of target genes in different cell types. One of the most commonly activated signaling pathways in PCa is the PTEN/PI3K pathway, which has been implicated in prostate carcinogenesis. Deletion and inactivating mutations of PTEN are events in human PCa occurrence and development. FOXO proteins play a pivotal role in a variety of biological processes, including apoptosis, the cell cycle, differentiation, stress responses, DNA damage repair and glucose metabolism [26]. Activation of FOXO subfamily members upregulate the cell-cycle inhibitors p21Cip1 [27] and p27Kip1 [28], and downregulate the cell cycle regulator cyclin D1/2, consequently leading to G1/S cell-cycle arrest. Therefore, the FOXO transcription factors are key tumor suppressors. Growing evidence suggests that activation of FOXO1induces apoptosis in prostate cancer cells.

Multiple miRNAs regulate PTEN, FOXO1 and SMAD2, resulting in cancer progression. SMAD2 is a novel target of miR-486-5p identified in this study. It was reportedly playing the direct effects of anti-tumorigenic and growth-suppressive on prostate cancer cell lines [29, 30]. But, some miR-486-5p target genes previously validated in other cellular systems (i.e., OLFM4, SIRT1, IGF1R, PIM-1) are absent in this study. It suggested that miR-486-5p regulates additional cell type-specific target genes. SAMD2 played a critical role in the basal epithelial or stem cell compartment of the prostate as a tumor suppressor. Our study found that miR-486-5p suppressed the Smad2/TGF-b signaling pathway activities in PCa.

Given the frequent activation of the PTEN/PI3K, Smad2/TGF-b and FoxO signaling pathways in PCa and their demonstrated tumor suppressor activity in clinical patients and mouse models, miR-486-5p-mediated regulation of these multiple pathways should play an important role in both PCa development and maintenance; hence, targeting these pathways has therapeutic value in treating PCa harboring miR-486-5p overexpression.

HIF-1a is the “master” regulator of gene expression in cellular response to hypoxia *in vitro* and ischemia *in vivo* [31]. HIF-1a mediated expression of erythropoietin, angiogenic factors, and glycolytic enzymes facilitates oxygen delivery to ischemic tissues and metabolic adaption of hypoxic cells to the low-oxygen environment [32]. HIF-1a overexpression under normoxia could serve as a biomarker for chemoresistance, radioresistance and castration resistance in prostate cancer [33]. Recent work has further suggested a reciprocal regulation between HIF-1a and miRNAs. On the one hand, HIF-1a exerts some of its effects through miRNAs, most notably miR-210 in various cancer and noncancer cell types [34–37]. Conversely, HIF-1a is also a regulatory target of several miRNAs, including miR-199a, −519c, and −20b and the miR-17-92 cluster [38–41].

TCF12 is believed to be a transcription factor and has been reported to have a close relationship with invasion of cancer [42]. The role of TCF12 in prostate cancer has not been reported yet. We found a TCF12 binding site located in the promotor region of miR-486-5p and the initial results suggested TCF12 could upregulate the expression of miR-486-5p via regulation of the promotor.

In conclusion, the current study has highlighted miR-486-5p as an important miRNA in the pathogenesis of PCa. MiR-486-5p was markedly induced via HIF-1a and TCF12 and repressed multiple pathways, including PTEN/PI3K, SMAD2/TGF-b and FoxO signaling pathways. These findings generate a gross profile for prostate tumorigenesis. Delineation of such process extends new insights of the pathogenesis of PCa and suggests a novel miRNA-based therapeutic approach.

## MATERIALS AND METHODS

### Patients

Formalin-fixed and paraffin-embedded tumor and adjacent normal tissues were from the archives of the Institutes for Pathology, Drum Tower Hospital, Medical School of Nanjing University. After all tissues were reviewed by two experienced pathologists, specimens including tumor and adjacent normal tissues from 23 patients undergoing radical prostatectomy were randomly selected, and the baseline characteristics of that population are listed in Supplementary Table 2 and further grouped by Gleason score (low risk:≤6, intermediate risk: 7 and high risk:≥8). The study was approved by the institutional review board of the Nanjing University School of Medicine, Nanjing, China and consistent with the Declaration of Helsinki.

### Cell culture

Three human prostate carcinoma cell lines (LNCaP, PC3, and Du145) and a nonmalignant epithelial prostate cell line (RWPE-1) were purchased from the Type Culture Collection of the Chinese Academy of Sciences, Shanghai, China. Prostate carcinoma cell lines were cultured in DMEM/F12 and supplemented with 10% (vol/vol) fetal bovine serum (FBS), 50 U/ml of penicillin and 50 mg/ml of streptomycin. RWPE-1 cell line was cultured in a keratinocyte serum free medium (K-SFM) supplemented with 0.05 mg/ml bovine pituitary extract and 5 ng/ml human recombinant epidermal growth factor 1-53. All cell lines were maintained in a humidified incubator at 37°C and 5% CO_2_.

For PC-3 and DU145 cells, the differences among three groups, transfected with miR-486-5p inhibitor (IH-miR), inhibitor negative control (NC-miR) and only transfection reagents (Mock) were observed in the order presented. For RWPE-1 cells, differences among the three similar groups, transfected with MiR-486-5p mimics (MI-miR), negative control (NC-miR) and only transfection reagents (Mock) were observed.

### Animal study

Male nude mice approximately 4 to 6 weeks old were purchased from the Model Animal Research Center of Nanjing University. Around 1×10^7^ DU145 cells, transfected with synthesized miR-486-5p inhibitor (IH-miR) or inhibitor negative control (NC-miR), were subcutaneously injected into two flanks of these nude mice (n=10), respectively. The small (S) and large (L) diameters of the lesion were measured at every 4 to 5 day interval, and tumor volume was calculated using the formula S^2^×L/2. After eight weeks, all animals were sacrificed by CO_2_. The tumor xenografts were immediately harvested and measured in pairs. Each tumor was divided into two parts, one fixed in formalin and another kept in −80°C until use. All the procedures of animal administration were approved by the Ethics Committee for Animal Research of Nanjing University.

### Plasmid construction

To generate Smad2 luciferase reporter constructs, the 3′ UTRs with the miR-486-5p binding sites were synthesized and cloned into GP-miRGLO vector which have both firefly and Renilla luciferase reporter at *Sac*I and *Xho*I restriction site (Promega, USA). The cloned sequences of wild-type (WT) and mutation (MUT) are listed in Supplementary Table 3. To generate recombinant vector for overexpression of Smad2, HIF-1a and TCF12, the coding sequence of wild-type Smad2 was cloned into p-CMV5B at *Sal*I and *Sma*I restriction site (Promega, USA), while the coding sequence of wild-type HIF-1a and TCF12 were both cloned into GV219 at *Xho*I and *Kpn*I. The empty vectors were used as a control. All constructs were sequenced to verify integrity.

### *In situ* hybridization (ISH)

Slides with sections (3 μm) of tissue specimens enrolled were stained using double digoxigenin (DIG)-labeled, miRCURY LNA™ (locked nucleic acid)-based detection probes. The probes specific for miR-486-5p were 5′CTCGGGGCAGCTCAGTACAGGA3′, U6 (5′CACG AATTTGCGTGTCATCTT3′, Exiqon) as inter control, and scramble-miR (5′GTGTAACACGTCTATACGCCCA3′) as negative control. After hybridization, slides were viewed with a Nikon Eclipse 80i microscope equipped with a digital camera and images were analyzed by Image-Pro Plus software, the semi-quantitative expression of miR-486-5p was normalized to U6 levels.

### GEO database analysis

After entering “prostate cancer” “microRNA” OR “miRNA,” we chose the data related to microRNA expression in prostate cancer from Gene Expression Omnibus (GEO) data sets (www.ncbi.nlm.nih.gov), and six groups of mircroRNA expression profiles (Supplementary Table 4) data related to expression of miR-486-5p in tissue were acquired. After normalization, these data were analyzed with SPSS and RevMan software.

### Cell viability assay

Cell viability was assessed with proliferation assay, cell cycle analysis, migration and Matrigel invasion assay and colony formation assay [10]. The proliferating capacity of IH-miR, NC-miR and mock cells was assessed on viable cells, which were counted with trypan blue. Cell-cycle progression of the three groups was examined with a Cell Cycle Detection Kit (KeyGEN, China) and performed on FACSCalibur (BD Biosciences, USA). Migratory and invasive ability was assessed using commercial Matrigel and control transwell chambers. The migrated cells were stained with crystal violet solution, dissolved in 33% ethanoic acid buffer, and then measured at OD570 nm for quantification. Colony formation assay was assessed by counting the clone numbers. Cell clones were stained with crystal violet solution, dissolved in 33% ethanoic acid buffer, and then measured at OD570 nm for quantification.

Cell wound healing assay was performed to assess the migration capacity. In detail, cells were seeded in 6-well plates with 5 × 10^5^ cells per well and cultured with different mediums. Then, a wound was made by using a 100 μ l pipette tip on cell monolayer and photographs were taken at appropriate time to estimate the area occupied by migratory cells.

### RNA sequencing

Total RNA was extracted from cells by Trizol reagent (Invitrogen, USA). The complementary DNA (cDNA) libraries for single-end sequencing were prepared using Ion Total RNA-Seq Kit v2.0 (Life Technologies, USA) per the manufacturer's instructions through mapping of single-end reads. Before read mapping, clean reads were obtained from the raw reads by removing the adaptor sequences, reads with > 5% ambiguous bases (noted as N) and low-quality reads containing more than 20 percent of bases with qualities of < 13. The clean reads were then aligned to the human genome (version: GRCH38) using the MapSplice program (v. 2.1.6). In alignment, preliminary experiments were performed to provide the largest information on the AS events. Pathway analysis was used to find out the significant pathway of the differential genes based on the KEGG database. We turned to Fisher's exact test to select the significant pathway, and the threshold of significance was defined by the P-value and false discovery rate (FDR).

### Target analysis

Both Target Scan (www.targetscan.org/), and miRanda (http://www.microrna.org/ microrna/home.do) were used to predict the target gene of miR-486-5p. Conserved miRNA with a “good mirSVR score” refers to miRNA targets with ≤ − 0.1 score obtained from the support vector regression algorithm; mirSVR, were chosen as the potential target gene.

### Quantitative real-time PCR (qRT-PCR) analysis

After total RNA extraction, the TaqMan MicroRNA Reverse Transcription Kit (ABI, USA) was used for reversion with the specific reverse transcription primer (001278, ABI, USA). QRT-PCR was then conducted to evaluate the expression of miR-486-5p by the TaqMan (2×) Universal PCR Master Mix (No AmpErase UNG) (ABI, USA). The results were normalized to U6 levels. The mRNA levels of miR-486-5p potential targets, HIF-1a and TCF12 were also measured with qPCR. The primers were list in Supplementary Table 5. The total RNA was reverse transcribed to cDNA using the HiScript Q-RT SuperMix (Vazyme, China) and then real-time PCR was performed in the ABI StepOne Sequence Detection System (PE Applied Biosystems, Foster City, CA). All PCRs were performed in triplicate, and data analysis was performed using the ΔΔCT method.

### Dual luciferase reporter assay

To confirm the direct binding of miR-486-5p to the 3′ UTRs of Smad2, Du145 cells were transfected in 24 well plates with the wild-type (WT) or mutant reporter plasmid vector plus inhibitor or inhibitor negative control by Lipofectamine™ 2000. Twenty-four hours after transfection, the cells were harvested. Renilla and firefly luciferase activity were measured with the Dual Luciferase Reporter Assay kit (Promega, Madison, WI, USA) in GloMax 96 (Promega, USA). Relative luciferase activities were calculated after normalizing the luciferase activity of each experimental sample.

### Western blot analysis

Cells were washed twice in ice-cold PBS, and then solubilized in RIPA buffer (Beyotime, China). 30 μg of protein was separated on 10% polyacrylamide gels and transferred to Immobilon-P PVDF membrane with 0.45 um pore size (Millipore, Billerica, MA). After blockage in Tris- buffered saline with 0.1% Tween 20 (TBST) with 5% skim milk and 0.5% bovine serum albumin (BSA), the PVDF membranes were incubated with the specific primary antibody of the checked proteins overnight at 4°C. The membranes were washed with TBST 3 times and incubated with a secondary antibody, IgG, conjugated to HRP (Abbkine, USA) for two hours at room temperature. Signals were detected with chemiluminescent HRP substrate (Millipore, Billerica, MA). PTEN, FOXO1, Akt, phospho-Akt (Ser473), Phospho-Smad2 (Ser465/467) were purchased from Cell Signaling Technology (USA); Smad2 and GAPDH were purchased from Abcam.

### Immunohistochemistry (IHC)

Tumor xenografts were fixed in formalin and embedded in paraffin. Three-μm thick sections were used for immunohistochemical staining. PTEN and Ki-67 expression were assessed by evaluating the proportion and intensity of positively stained carcinoma cells. After inhibition of endogenous peroxidase activities for 30 min with methanol containing 0.3% H2O2, the sections were blocked with 2% BSA for 30 min and incubated overnight at 4°C with primary polyclonal rabbit anti-PTEN (Cell Signaling Technology, USA) and anti-Ki67 (Cell Signaling Technology, USA). After washing thrice with PBS, the slides were incubated with a secondary antibody for 1 h, followed by reaction with diaminobenzidine and counterstaining with Mayer's hematoxylin. Slides were viewed with a Nikon Eclipse 80i microscope equipped with a digital camera (DS-Ri1, Nikon, Shanghai, China).

### Statistical analysis

Data from GEO website were analyzed the by meta-analysis program, RevMan, version 5.0. All other statistical analyses were carried out using the statistical program SPSS, version 17.0. Statistical significance was determined with unpaired student t tests, except as noted for analyses of microarray data, which were examined with Fisher exact tests. P values less than 0.05 were considered statistically significant.

## SUPPLEMENTARY MATERIALS FIGURE AND TABLES



## Abbreviations

UTR: untranslated region
FBS: fetal bovine serum
IH-miR: miR-486-5p inhibitor
NC-miR: inhibitor negative control
WT: wild-type
MUT: mutation
LNA: locked nucleic acid
GEO: Gene Expression Omnibus
TBST: Tris buffered saline with Tween-20
BSA: bovine serum albumin
