# Supplementary material for: The miR-486-5p plays a causative role in prostate cancer through negative regulation of multiple tumor suppressor pathways
Source: Oncotarget. 2017 Aug 24;8(42):72835–46. doi: 10.18632/oncotarget.20427 (PMC5641172; doi:10.18632/oncotarget.20427)
Supplement: Supplementary file 1 [file oncotarget-08-72835-s001.pdf]

The miR-486-5p plays a causative role in prostate cancer through negative regulation of multiple tumor suppressor pathways

SUPPLEMENTARY MATERIALS

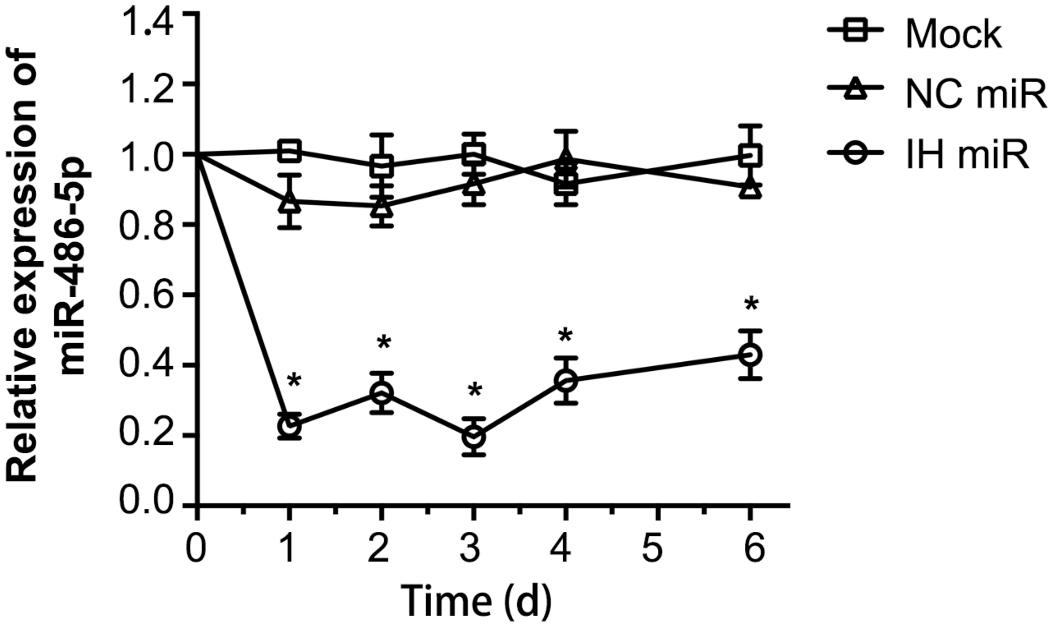

Supplementary Figure 1: The expression of mir-486-5p was maintain at low level for at least 6 days after transfected with inhibitor. The mir-486-5p specific inhibitor and inhitor negative control were transfected into Du145 cells and the expression of mir-486-5p was validated at different days by real-time PCR using U6 as control.

Supplementary Table 1: The relative expression of miR-486-5p (cancer tissue/ adjacent normal tissues) measured by ISH in different Gleason score cancer (p>0.05)

Patients number	Age (y)	tPSA (ng/ml)	Gleason score	Stage (TNM)
1	72	77.10	9	T2N1M0
2	61	12.3	7	T2cN0M0
3	74	22.04	7	T2cN0M0
4	78	6.46	6	T2cN0M0
5	76	10.4	7	T2cN0M0
6	65	64	7	T2N0M0
7	71	22.5	9	T3bN1M0
8	77	11.4	7	T2cN1M0
9	79	100.1	9	T3bN1M0
10	77	15.2	8	T3bN0M0
11	68	27.5	8	T3bN1M0
12	77	21.92	7	T2cN0M0
13	60	5.06	9	T2N0M0
14	63	21.02	7	T2N0M0
15	65	8.65	6	T2N0M0
16	77	13.81	7	T2cN0M0
17	72	11.6	6	T2cN0M0
18	70	27.9	8	T3bN0M0
19	62	7.6	6	T2N0M0
20	67	12.8	6	T2N0M0
21	57	24.3	7	T2N0M0
22	70	18.3	7	T2N0M0
23	65	33.9	8	T2N0M0

Supplementary Table 2: Clinical characteristics (age, tPSA, gleason score and stage) of the 23 patients whose specimen were chosen to be identified by ISH for mir-486-5p expression

Gleason score	6(n=5)	7(n=10)	≥8(n=8)
Relative expression	2.569±0.77	2.924±0.81	2.777±0.83

Supplementary Table 3: The clonde sequence of the luciferase reporter plasmid

luciferase reporter plasmid	Cloned sequence
SMAD2 WT	GAGCTCTCCCAAAGGTTTATTAATAACAGTAGTAGTTATGTGTACAGGTAATGTATCATCTCGAG
SMAD2 MUT	GAGCTCTCCCAAAGGTTTATTAATAACAGTAGTAGTTATGTCATGTCCTAATGTATCATCTCGAG

Supplementary Table 4: 6 groups mircroRNA expression profiles from GEO Datasets

1	GSE45604 (http://www.ncbi.nlm.nih.gov/geo/query/acc.cgi?acc=GSE45604)<br>Samples: 50 prostate cancer, 10 controls<br>Platform: GPL14613, Affymetrix Multispecies miRNA-2.0 Array
2	GSE23022 (http://www.ncbi.nlm.nih.gov/geo/query/acc.cgi?acc=GSE23022)<br>Samples: 20 prostate cancer, 20 controls<br>Platform: GPL8786, Affymetrix miRNA-1.0 Array
3	GSE36802 (http://www.ncbi.nlm.nih.gov/geo/query/acc.cgi?acc=GSE36802)<br>Samples: 21 prostate cancer, 21 controls<br>Platform: GPL8786, Affymetrix miRNA-1.0 Array
4	GSE21036 (http://www.ncbi.nlm.nih.gov/geo/query/acc.cgi?acc=GSE21036)<br>Samples: 113 prostate cancer, 28 controls<br>Platform: GPL8227, Agilent-019118 Human miRNA Microarray 2.0 G4470B
5	GSE8126 (http://www.ncbi.nlm.nih.gov/geo/query/acc.cgi?acc=GSE8126)<br>Samples: 60 prostate cancer, 16 controls<br>Platform: GPL5180, MRA-1001 miRHuman 16 (LC science)
6	GSE54010 (http://www.ncbi.nlm.nih.gov/geo/query/acc.cgi?acc=GSE54010)<br>Samples: 14 prostate cancer, 5 controls<br>Platform: GPL15159, Agilent-031181 Unrestricted Human miRNA V16.0 Microarray 030840

Supplementary Table 5: Primers sequence of the potential target genes for qRT-PCR

Gene symbol	Primer sequence
PTEN	F:5’ TTTGAAGACCATAACCCACCAC3’
	R:5’ ATTACACCAGTTCGTCCCTTTC3’
FOXO1	F:5’ GGATGTGCATTCTATGGTGTACC3’
	R:5’ TTTCGGGATTGCTTATCTCAGAC3’
TP53INP1	F:5’ TTCCTCCAACCAAGAACCAGA 3’
	R: 5’ GCTCAGTAGGTGACTCTTCACT 3’
SMAD2	F:5’ CCGACACACCGAGATCCTAAC3’
	R:5’ GAGGTGGCGTTTCTGGAATATAA3’
CDKN2B	F: 5’ AATTCCATTTTCGAAGCC 3’
	R:5’ CACAATGGAGCTAGAAGCA 3’
CDK4	F: 5’ATGGCTACCTCTCGATATGAGC 3’
	R:5’ CATTGGGGACTCTCACACTCT 3’
ARID4B	F: 5’ TTGATGGTGCATATCAGGAAGC 3’
	R:5’ TCAGTGTCTTCTCATCTCCGTC 3’
CTNNB1	F: 5’ CATCTACACAGTTTGATGCTGCT 3’
	R:5’ GCAGTTTTGTCAGTTCAGGGA 3’
ATR	F: 5’ TCCCTTGAATACAGTGGCCTA 3’
	R:5’ TCCTTGAAAGTACGGCAGTTC 3’
APC	F: 5’ AAAATGTCCCTCCGTTCTTATGG 3’
	R:5’ CTGAAGTTGAGCGTAATACCAGT 3’
CDK1	F: 5’ GGATGTGCTTATGCAGGATTCC 3’
	R:5’ CATGTACTGACCAGGAGGGATAG 3’
TNC	F: 5’ GCCCCTGATGTTAAGGAGCTG 3’
	R:5’ GGCCTCGAAGGTGACAGTT 3’
TCF12	F:5’ CCAGTAGTTATGGCAACCTTCAT3’
	R: 5’GACTCGTGTTTATGTCTGTTGGT3’
HIF-1a	F5’ ATCCATGTGACCATGAGGAAATG3’
	R5’ TCGGCTAGTTAGGGTACACTTC3’
